# Automated and Interviewer-Administered Mobile Phone Surveys in Burkina Faso: Sociodemographic Differences Among Female Mobile Phone Survey Respondents and Nonrespondents

**DOI:** 10.2196/17891

**Published:** 2020-07-14

**Authors:** Abigail R Greenleaf, Aliou Gadiaga, Yoonjoung Choi, Georges Guiella, Shani Turke, Noelle Battle, Saifuddin Ahmed, Caroline Moreau

**Affiliations:** 1 ICAP at Columbia University New York, NY United States; 2 Institut Supérieur des Sciences de la Population University of Ouagadougou Ouagadougou Burkina Faso; 3 iSquared, Information x Insight Severna Park, MD United States; 4 Department of Population, Family and Reproductive Health Johns Hopkins University Baltimore, MD United States; 5 Soins et santé Center for Research in Epidemiology and Population Health INSERM Villejuif France

**Keywords:** cell phone, mHealth, Africa South of the Sahara, Burkina Faso, methodology, survey, nonrespondents, survey methods, interviews, telephone

## Abstract

**Background:**

The remarkable growth of cell phone ownership in low- and middle-income countries has generated significant interest in using cell phones for conducting surveys through computer-assisted telephone interviews, live interviewer-administered surveys, or automated surveys (ie, interactive voice response).

**Objective:**

This study aimed to compare, by mode, the sociodemographic characteristics of cell phone owners who completed a follow-up phone survey with those who did not complete the survey.

**Methods:**

The study was based on a nationally representative sample of women aged 15 to 49 years who reported cell phone ownership during a household survey in Burkina Faso in 2016. Female cell phone owners were randomized to participate in a computer-assisted telephone interview or hybrid interactive voice response follow-up phone survey 11 months after baseline interviews. Completion of the phone survey was defined as participants responding to more than 50% of questions in the phone survey. We investigated sociodemographic characteristics associated with cell phone survey completion using multivariable logistic regression models, stratifying the analysis by survey mode and by directly comparing computer-assisted telephone interview and hybrid interactive voice response respondents.

**Results:**

A total of 1766 women were called for the phone survey between November 5 and 17, 2017. In both the computer-assisted telephone interview and hybrid interactive voice response samples, women in urban communities and women with secondary education or higher were more likely to complete the survey than their rural and less-educated counterparts. Compared directly, women who completed the hybrid interactive voice response survey had higher odds of having a secondary education than those who completed computer-assisted telephone interviews (odds ratio 1.7, 95% CI 1.1-2.6).

**Conclusions:**

In Burkina Faso, computer-assisted telephone interviews are the preferred method of conducting cell phone surveys owing to less sample distortion and a higher response rate compared with a hybrid interactive voice response survey.

## Introduction

### Background

Cell phone ownership in sub-Saharan Africa (SSA) has quadrupled over the past 10 years, rising to 444 million unique subscribers in 2017 [1]. Concomitant to increased cell phone ownership in SSA is a rise in the use of cell phones for remote data collection [2]. One of the first attempts to create a nationally representative estimate from an interactive voice response (IVR) survey in SSA took place in Ethiopia, Mozambique, and Zimbabwe in 2015 [3]. Since this study, phone survey research expanded and includes the use of computer-assisted telephone interviews (CATIs) and SMS surveys, but the optimal mode for nationally representative cell phone surveys is yet to be identified.

A CATI involves a live interviewer who administers the full survey, usually in a call center. In contrast, an IVR survey is conducted without an interviewer and requires that the respondents use their keypad to answer a prerecorded question or prompt (eg, “If yes, press 1. If no, press 2”). A hybrid IVR survey is a modified version of an IVR survey: a live interviewer opens the call, confirms respondent eligibility, administers consent, and explains how to respond to an IVR question before transferring the respondent to the IVR survey. Finally, an SMS approach asks the respondent to answer questions via a text message [4].

Although these remote modes hold the possibility of more rapid, cost-effective data collection, a transition from face-to-face (FTF) survey to cell phone data collection raises issues of survey quality. To operationalize survey quality, survey researchers devised the concept of total survey error, comprising 5 components: frame, nonresponse, specification, measurement, and data processing errors [5,6].

This analysis addresses nonresponse error, which occurs when people who are sampled but not interviewed differ in a non-negligible way from those who are interviewed [5]. In telephone surveys, nonresponse generally stems from 3 causes: (1) failure to contact sampled respondents, (2) refusal to participate, and (3) ability or language constraints [7]. Nonresponse bias occurs when the outcome of interest is systematically different between people who are sampled but who do not complete the survey and those who complete the survey [5,8].

Although there is a well-established body of literature on causes of error in phone surveys in high-income countries [9,10], nonresponse error and bias in cell phone surveys have been minimally explored in SSA because of the recency of the approach in the continent. Among the few phone survey studies conducted in low- and middle-income countries (LMICs), the representativeness of a sample is rarely considered. Rather, studies have mainly addressed the feasibility and measurement error [11]. Studies that attempt to profile nonrespondents often assess sample distortion by comparing phone survey respondents with a reference population based on census or Demographic and Health Survey (DHS) data or comparing early and late responders [6,12,13]. Moreover, 2 recent studies in SSA (Ghana: IVR and Cote d’Ivoire: CATI) found that completers of cell phone surveys were more likely to be young, urban, educated, and male compared with distributions in a representative sample survey [12,13]. Although comparing a phone study sample and a DHS sample can provide some insight on nonrespondents, the comparison cannot distinguish a frame error (cell phone ownership) from a nonresponse error (not answering the survey phone call among people who own phones) because the sample frame is unknown. The paucity of remote data collection studies in SSA and, in particular, studies using a known representative sampling frame to assess nonresponse bias is a notable knowledge gap.

To address this gap, we conducted a study in Burkina Faso using the Performance Monitoring and Accountability 2020 (PMA2020) platform. Since 2014, the PMA2020 platform has annually tracked family planning indicators and evaluated the impact of specific family planning programs using FTF surveys among a nationally representative probabilistic sample. The PMA2020 survey has completed 5 rounds of data collection in Burkina Faso and is exploring new approaches, including cell phone surveys, to collect quality data at a lower cost [6,11,12] The 2016 PMA2020 survey in Burkina Faso found that 86% of households owned a cell phone and 47% of women reported personal cell phone ownership [14].

### Objectives

The objective of this research was to identify sociodemographic characteristics related to phone survey completion among a representative sample of female phone owners in Burkina Faso. We examined these questions for 2 phone survey modes: CATI and hybrid IVR.

## Methods

### Study Design

The study used a nationally representative sample of women of reproductive age, who owned a cell phone, identified in the Burkina Faso PMA2020 Round 4 (R4) survey. These female cell phone owners were randomized to receive a phone follow-up survey using either a CATI or a hybrid IVR. Hybrid IVR was preferred over IVR because it was hypothesized that having a live interviewer introduce the survey would increase response rates and improve data quality, given that only 30% of women in Burkina Faso are literate [15]. Phone follow-up occurred 11 months after the PMA2020 R4 FTF survey. The phone survey was a shortened version of PMA2020’s standard FTF questionnaire.

### Participants

**Figure 1:**
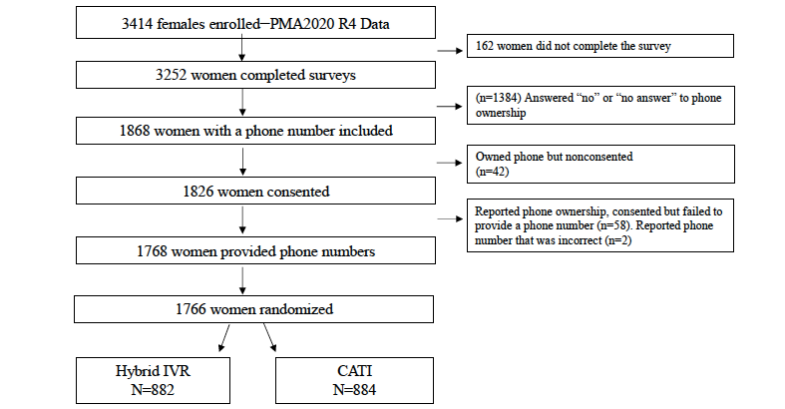
Study enrollment flow chart.

The PMA2020 R4 survey in Burkina Faso used a two-stage stratified cluster design, starting with a selection of 83 enumeration areas using probability proportional to size sampling. The enumeration areas were stratified by rural or urban geographies; then, 35 of the approximately 200 households within each enumeration area were randomly selected. Within each sampled household, all women aged 15 to 49 years were eligible to participate, and those who provided written consent were included. Women who met the FTF inclusion criteria, had a cell phone, consented to be contacted for a follow-up survey, and provided a phone number were eligible for follow-up. Of the 3215 female respondents interviewed during PMA2020 R4 (95.4% response rate), 1839 (57.20%) owned a cell phone; among them, 1766 (96.03%) consented to be contacted again for this study (Figure 1). The aforementioned 1766 women were assigned a random number in Stata (using the *generate random* command; StataCorp 2017) and then sorted by random numbers (from smallest to largest). Women were divided into 6 language groups (5 languages and one *other language* category) and then divided again into groups of approximately 120 women, assigned to an arm (hybrid IVR: n=882 or CATI: n=884), and then finally assigned to an interviewer. During the phone follow-up, women heard an abbreviated standardized verbal disclosure (consent) of key study information that emphasized the voluntary nature of the survey, then consented or refused.

### Study Procedures

A total of 15 trained interviewers conducted the phone follow-up survey, operating from a call center in Ouagadougou 7 days a week between 8 AM and 8 PM. Interviewers called all eligible women (consenting female cell phone owners from PMA2020 R4).

Respondents were called up to 6 times at varying times of the day and at least once during the weekend. If a respondent answered and wanted to be called back within 15 min, the operator could accommodate the request, otherwise the respondent was called back the next day. A respondent was not called back if she refused to consent to the study or if someone answered the call who did not know the respondent. All respondents saw the same phone number appearing on their cell phone, which was identifiable as a landline phone number. The call center phone number could not be called back. Women who completed the survey were sent the equivalent of US $1 phone credit the day after completing the interview.

### Questionnaire

The phone surveys (CATI and hybrid IVR) used the same question wording as the PMA2020 FTF questionnaire with only minor modifications. They included 17 questions: 5 introductory questions to identify the respondent, 4 demographic questions (age, area of residence, marital status, and parity), 5 questions about the respondent’s awareness of modern contraceptive methods (intrauterine device, implant, condom, pills, and injectables), and 3 questions on contraceptive use (current use, previous method used, or pregnancy intention). After consent was administered, women receiving the CATI were asked if the interviewer could call the participant back should the call drop. For women receiving the hybrid IVR survey, after consent, they heard an explanation of what to expect during an IVR survey and then were asked to press 1 on their keypad. If the respondent was unable to press 1, she was considered incapable of participating in the IVR survey, and the interview ended. If the woman pressed 1, she was transferred to a prerecorded voice providing instructions about how to repeat or skip a question. Then, she was asked a practice question about the country that she currently lives in. From this point on, the questionnaires for both modes were identical until the last question. The last question in the CATI asked the respondent which region she lived in, and the last IVR question asked the respondent to enter her age.

Of the 17 survey questions, 1 question was numeric (age), 3 were multiple choice, and 12 were binary with a yes or no response option. The key question “What are you or your partner currently using to delay or avoid a pregnancy?” was field coded by interviewers in the CATI, and was presented as a multiple-choice question with 6 response options in the IVR survey. For the IVR survey, the school question was divided into 2 questions: first, asking if the respondent had ever been to school, and second, if the respondent answered affirmatively, asking her up to what level she had studied. Both the CATI and hybrid IVR surveys were offered in 5 languages. Each respondent received a call in the language used in her PMA2020 FTF interview. The questionnaire was written in French in the software, and CATI interviewers translated in real time, after having developed and refined consistent translations in the local language during the training.

### Ethical Approval

Both the FTF PMA2020 survey and phone survey received institutional review board approval from the Johns Hopkins Bloomberg School of Public Health and from the ethical committee in Burkina Faso, *Comité d’éthique pour la recherche en santé*.

### American Association for Public Opinion Research Measures

Using the American Association for Public Opinion Research (AAPOR) call disposition codes [16] and study-specific considerations, we defined 8 respondent categories. All participants were eligible and were categorized as respondents or nonrespondents. Our outcome of interest was *completing the phone survey*, defined as respondents answering 50% or more of the relevant survey questions (eg, those assigned partial and complete call disposition codes). The follow-up data were only used to classify respondents into 8 groups (disposition codes) and thus calculate survey outcome rates.

### Respondent Characteristics Measures

Sociodemographic information was collected during PMA2020 R4 and therefore available for all eligible women, regardless of whether the woman responded to the phone follow-up survey. Sociodemographic variables included age, categorized into 5-year age groups for descriptive analysis and into 4 age groups for logistic regressions (15-19, 20-29, 30-39, and 40-49 years). Additional covariates were current union status (in union—ie, currently married or living with a partner vs not in union), residential area (urban vs rural), highest grade level attended (none, primary, and secondary or higher), household wealth (lowest, middle, and highest tertiles), having electricity (yes vs no), and parity (ever given birth vs never given birth). Household wealth in PMA2020 surveys is a summary measure of the relative wealth of a household in the country based on household assets and housing conditions. The indicator is constructed using a principal component analysis, which is widely used in population-based surveys such as DHS surveys [17].

### Statistical Analysis

To examine whether randomized groups (women assigned to the CATI vs hybrid IVR survey) were similar according to their background characteristics at baseline (PMA2020 R4 survey), we explored the distribution of selected demographic and socioeconomic variables by mode. We used American Association for Public Opinion Research call disposition classifications [16] to classify respondents and calculated 4 AAPOR’s phone survey outcome rates (Table 1).

**Table 1:** Final disposition code by study arm (computer-assisted telephone interview vs hybrid interactive voice response) among female cell phone owners in Burkina Faso.

Participants, American Association for Public Opinion Research categorization^a^, and explanation			Hybrid interactive voice response (n=882), n (%)	Computer-assisted telephone interview (n=884), n (%)
**Nonrespondents**				
	NC^b^ (2.20)	Noncontact (did not pick up)	225 (25.5)	228 (25.8)
	O^c^ (2.36)	Noncontact (someone picked up the phone call, but interviewer never spoke with the woman)	173 (19.6)	134 (15.2)
	R^d^ (2.12)	Refusal preconsent	90 (10.2)	54 (6.1)
	R (2.111)	Refusal	14 (1.6)	6 (0.7)
	R (2.121)	Break-off interactive voice response	31 (3.5)	N/A^e^
	R (2.12)	Break-off (consented but <50% of relevant questions answered)	174 (19.7)	17 (1.9)
**Respondents**				
	P^f^ (1.2)	Partial (50%-80% of relevant questions answered)	18 (2.0)	5 (0.6)
	I^g^ (1.1)	Complete (more than 80% of relevant questions answered)	157 (17.8)	440 (49.8)

^a^Final disposition codes for random digit dial telephone surveys.

^b^NC: noncontact.

^c^O: other.

^d^R: refusal and break-off.

^e^N/A: not applicable.

^f^P: partial interview.

^g^I: complete interview.

Analyses were stratified by mode of data collection (CATI and hybrid IVR). All eligible women were included in the analysis. We compared the distribution of the sociodemographic characteristics between completers and noncompleters using chi-square tests. We then conducted multivariable logistic regression models to identify the independent factors associated with survey completion. We fitted the models separately for each survey mode (hybrid IVR and CATI). We did not include marital status or parity in the multivariable models because these characteristics were not significantly related to completion in the bivariate analyses. Electricity was also not included because of its high correlation with wealth. Finally, using multivariable logistic regression, we pooled hybrid IVR and CATI survey completers to compare the distribution of survey mode by background characteristics to evaluate whether background characteristics were different by mode. All analyses were conducted using Stata version 15.

## Results

### Respondent Characteristics

The average age of the 1766 women enrolled in this study—all of whom were contacted by phone at least once between November 5 and 17, 2017—was 28.5 years (SD 8.9) (Table 2). Overall, 42.02% (742/1766) of women had never attended school, 19.85% (351/1766) had attended primary school, and 38.12% (674/1766) had secondary or higher education. Of these, 65.27% (1154/1766) were married and 71.91% (1270/1766) were parous. Moreover, 66.57% (1177/1766) of the women were living in rural areas and 61.99% (1096/1766) had electricity in the household. Finally, 66.57% (1177/1766) were in the highest wealth tertile, with 18.10% (320/1766) in the middle tertile and 15.33% (271/1766) in the lowest tertile. After randomization, women in the hybrid IVR and CATI groups were similar across all sociodemographic characteristics examined in this study (Table 2).

**Table 2:** Sample characteristics of female cell phone owners in Burkina Faso, overall and by study arm.

Characteristics			Total study population (N=1766)	Hybrid interactive voice response arm (n=882)	Computer-assisted telephone interview arm (n=884)
**Age** **(years)**					
	Mean (SD)		28.5 (8.9)	28.6 (9.1)	28.4 (8.7)
	**Range, n (%)**				
		15-19	338 (19.12)	163 (18.5)	175 (19.8)
		20-24	351 (19.85)	175 (19.8)	175 (19.8)
		25-29	342 (19.34)	176 (19.9)	166 (18.8)
		30-34	265 (14.99)	126 (14.3)	139 (15.7)
		35-39	207 (11.71)	192 (11.6)	105 (11.9)
		40-44	158 (8.94)	82 (9.3)	76 (8.6)
		45-49	107 (6.05)	58 (6.6)	48 (5.4)
**Residential area, n (%)**					
		Urban	591 (33.43)	294 (33.3)	295 (33.4)
		Rural	1177 (66.57)	588 (66.7)	589 (66.6)
**Marital status, n (%)**					
		Currently not in union	614 (34.73)	312 (35.4)	301 (34.1)
		Currently in union	1154 (65.27)	568 (64.4)	583 (65.9)
**Highest school attended, n (%)**					
		Never	743 (42.02)	370 (42.0)	370 (41.9)
		Primary	351 (19.85)	181 (20.5)	170 (19.2)
		Secondary or higher	674 (38.12)	330 (37.4)	344 (38.9)
**HH^a^ wealth (tertile), n (%)**					
		Lowest	271 (15.33)	137 (15.5)	133 (15.1)
		Middle	320 (18.10)	155 (17.6)	165 (18.7)
		High	1177 (66.57)	590 (66.9)	586 (66.3)
**Parity, n (%)**					
		Yes	1270 (71.91)	639 (72.5)	630 (71.3)
		No	496 (28.08)	243 (27.5)	254 (28.7)
**HH electricity, n (%)**					
		Yes	1096 (61.99)	548 (62.1)	548 (62.0)
		No	672 (38.01)	334 (37.9)	336 (38.0)

^a^HH: household.

### American Association for Public Opinion Research Outcomes

The percentage of eligible women who did not answer any of the 6 calls was the same among the 2 modes (hybrid IVR: 25.5% (225/882); and CATI: 25.8% (228/884); NC in Table 1). Overall, 47.11% (832/1766) women consented to participate in the phone interview when called: 43.1% (380/882) of women in the hybrid IVR arm and 52.2% (462/884) of women in the CATI arm (*P*<.001; AAPOR categories: R [2.121], R [2.12], *P* [1.2], and I [1.1] in Table 1). Break-off, defined as consenting but answering less than 50% of the questions, was substantially higher for the women in the hybrid IVR arm 23.2% (205/882) than those in the CATI arm 1.9% (17/884). Altogether, among all women randomized to the hybrid IVR arm, 19.8% (175/882) completed the survey (partial interviews: 18/882, 2.0%; complete interviews: 157/882, 17.8%), whereas 50.4% (445/884) of those randomized to the CATI arm completed the survey (partial interviews: 5/884, 0.6%; and complete interviews: 440/884, 49.8%).

The 4 essential AAPOR survey outcome rates (response, cooperation, refusal, and contact) based on disposition codes are presented in Table 3. The contact rate, the proportion of all cases in which a responsible member of the unit was reached by the survey, was similar between the 2 modes (54.8% for hybrid IVR and 59.1% for CATI). The refusal rate for hybrid IVR surveys (11.9%) was almost double the refusal rate for CATI (6.8%), although the difference was not statistically significant (*P*=.23). The cooperation rate, defined as completed interviews over contacted respondents, for CATIs (85.2%) dwarfs the cooperation rate for hybrid IVR surveys (35.9%; cooperation rate 2). Finally, the response rate, a measure of the number of partial or completed interviews over all eligible respondents, was twice as high in the CATI arm compared with the hybrid IVR arm. Specifically, the response rate among women in the CATI arm was 50.3%, compared with a response rate of 19.8% among women in the hybrid IVR arm (response rate 6).

**Table 3:** Follow-up phone call outcome rates by study arm (computer-assisted telephone interview vs hybrid interactive voice response) among female cell phone owners in Burkina Faso.

Outcome rates		Hybrid interactive voice response arm (n=882)	Computer-assisted telephone interview arm (n=884)
**Response rates**			
	Response rate 5: 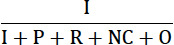	156 (17.7)	439 (49.7)
	Response rate 6: 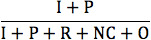	175 (19.8)	445 (50.3)
**Cooperation rates**			
	Cooperation rate 1: 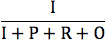	284 (32.2)	743 (84.0)
	Cooperation rate 2: 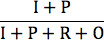	317 (35.9)	753 (85.2)
**Refusal rate**			
	Refusal rate 3: 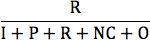	105 (11.9)	60 (6.8)
**Contact rate**			
	Contact rate 3: 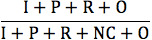	483 (54.8)	522 (59.1)

### Bivariate and Multivariable Logistic Regressions

In the bivariate analyses (Table 4), we found that among hybrid IVR respondents, survey completion was not dependent on age (*P*=.48) but was lower among women living in rural areas than those living in urban areas (27/294, 9.2% vs 148/588, 25%; *P*=.002). The proportion of women who completed the survey was lower among women with no education compared with those with secondary education or higher (46/371, 12.4% vs 94/330, 28.5%; *P*<.001). Twice as many women who had household electricity completed the survey compared with women who had no electricity in the household (133/548, 24.3% vs 42/334, 12.6%; *P*<.001), and 3 times as many women in the highest tertile completed the survey compared with those in the lowest tertile (12/137, 8.8% vs 146/590, 25%; *P*<.001).

The patterns of completion were similar for CATIs, with residential area, wealth tertile, and household electricity being associated with completion. However, education was unrelated to CATI completion 45.9%, (170/370) of those with no education vs 53.8% (185/344) of those with secondary education or higher; *P*=.84. On the other hand, survey completion was dependent on age, with only 33% of women aged 15 to 19 years completing the survey, compared with 45.1% to 63.3% of women aged 20 to 49 years (*P*<.001). The proportion of urban women who completed the survey was greater than the proportion of rural women who completed the survey (333/589, 56.5% vs 112/295, 38.0%; *P*<.001).

The results from the multivariable logistic regression models comparing women who completed and those who did not complete the hybrid IVR survey and CATI are presented in Table 5. Women who were aged 30 years and older were more likely to complete the hybrid IVR survey than women aged 15 to 19 years, as well as women with secondary education, who had 2.5 times the odds of completing the survey relative to women with no education (95% CI 1.6-3.9). Urban women had twice the odds of completing the survey compared with rural women (95% CI 1.1. 3.6).

The odds of CATI completion were elevated for women aged 20 years and older compared with teenagers. Women in urban communities were more likely to complete the CATI survey compared with their rural counterparts (odds ratio [OR] 1.7, 95% CI 1.1-2.5) as were women with a secondary education or more who had 40% higher odds of completing the survey than women with no education (OR 1.4, 95% CI 1.1-2.0).

In multivariable logistic regression among survey completers (n=620), which assessed the survey mode according to women’s background characteristics, the only significant difference was education: women who attended secondary or higher education were 70% more likely to be hybrid IVR completers than those who had no education (OR 1.7, 95% CI 1.1-2.6; Table 6).

**Table 4:** Percent completing the hybrid interactive voice response or computer-assisted telephone interview survey by women’s sociodemographic characteristics among female cell phone owners randomized to each arm.

Characteristics		Hybrid interactive voice response				Computer-assisted telephone interview		
		Participants who did not complete the survey	Participants who completed the survey	*P* value	Participants who did not complete the survey		Participants who completed the survey	*P* value
Total, n (%)		707 (80)	175 (20)	N/A^c^	439 (49)		445 (51)	N/A
**Age (years), n (%)**				.48				<.001
	15-19	137 (84.1)	26 (15.9)		116 (66.3)		59 (33.7)	
	20-24	137 (78.3)	38 (21.7)		96 (54.9)		79 (45.1)	
	25-29	145 (82.4)	31 (17.6)		66 (39.8)		100 (60.2)	
	30-34	94 (74.6)	32 (25.4)		55 (39.6)		84 (60.4)	
	35-39	84 (82.4)	18 (17.6)		56 (53.3)		49 (46.7)	
	40-44	65 (79.3)	17 (20.7)		28 (36.8)		48 (63.2)	
	45-49	45 (77.6)	13 (22.4)		22 (45.8)		26 (54.2)	
**Residential area, n (%)**				<.001				<.001
	Rural	267 (90.8)	27 (9.2)		183 (62.0)		112 (38.0)	
	Urban	440 (74.8)	148 (25.2)		256 (43.5)		333 (56.5)	
**Marital status, n (%)**				.28				.94
	Currently not in union	244 (78.2)	68 (21.8)		150 (49.8)		151 (50.2)	
	Currently in union	463 (81.2)	107 (18.8)		289 (49.6)		294 (50.4)	
**Highest school attended, n (%)**				<.001				.08
	Never	325 (87.6)	46 (12.4)		200 (54.1)		170 (45.9)	
	Primary	146 (80.7)	35 (19.3)		80 (47.1)		90 (52.9)	
	Secondary or higher	236 (71.5)	94 (28.5)		159 (46.2)		185 (53.8)	
**HH^a^ wealth (tertile), n (%)**				<.001				<.001
	Lowest	125 (91.2)	12 (8.8)		86 (64.7)		47 (35.3)	
	Middle	138 (89.0)	17 (11.0)		92 (55.8)		73 (44.2)	
	Highest	444 (75.3)	146 (24.7)		261 (44.5)		325 (55.5)	
**Parity, n (%)**				.50				.13
	No	200 (78.7)	54 (21.3)		143 (53.6)		124 (46.4)	
	Yes	507 (80.7)	121 (19.3)		296 (48.0)		321 (52.0)	
**HH electricity, n (%)**				<.001				.005
	No	292 (87.4)	42 (12.6)		187 (55.7)		149 (44.3)	
	Yes	415 (75.7)	133 (24.3)		252 (46.0)		296 (54.0)	

^a^HH: household.

**Table 5:** Odds of completing the phone follow-up survey by background characteristics: multivariable logistic regression analyses among the 2 study arms. Italics indicates *P*<.05.

Characteristics		Hybrid interactive voice response completers versus noncompleters (n=882), adjusted odds ratio (95% CI)	Computer-assisted telephone interview completers versus noncompleters (n=884), adjusted odds ratio (95% CI)
**Age group (years)**			
	15-19	Reference	Reference
	20-29	1.4 (0.8-2.3)	*2.3 (1.5-3.4)*
	30-39	*2.1 (1.2-3.8)*	*2.7 (1.8-4.2)*
	40-49	*2.1 (1.1-4.0)*	*3.8 (2.3-6.4)*
**Residential area**			
	Rural	Reference	Reference
	Urban	*2.0 (1.1-3.6)*	*1.7 (1.1-2.5)*
**Highest school attended**			
	No education	Reference	Reference
	Primary	1.5 (0.9-2.4)	1.3 (0.9-1.9)
	Secondary or more	*2.5 (1.6-3.9)*	*1.4 (1.1-2.0)*
**Household wealth (tertile)**			
	Lowest (reference all other groups)	0.8 (0.4-1.8)	0.7 (0.4-1.2)
	Highest (reference all other groups)	1.3 (0.7-2.5)	1.1 (0.7-1.7)

**Table 6:** Odds of completing the hybrid interactive voice response survey compared with completing the computer-assisted telephone interview survey by background characteristics: multivariable logistic regression analyses. Italics indicates *P* value below .05.

Characteristics		Hybrid interactive voice response versus computer-assisted telephone interview among survey completers (N=620; reference group: CATI), adjusted odds ratio (95% CI)
**Age group (years)**		
	15-19	Reference
	20-29	0.9 (0.5-1.5)
	30-39	0.9 (0.5-1.7)
	40-49	1.1 (0.5-2.1)
**Residential area**		
	Rural	Reference
	Urban	1.4 (0.8-2.4)
**Highest school attended**		
	No education	Reference
	Primary	1.3 (0.8-2.2)
	Secondary or more	*1.7 (1.1-2.6)*
**Household** **wealth (tertile)**		
	Lowest (reference: all other groups)	1.1 (0.5-2.5)
	Highest (reference: all other groups)	1.4 (0.8-2.7)

## Discussion

This analysis offers 3 main findings. First, we found that the CATI response and cooperation rates were more than double the rates for hybrid IVR surveys because of high break-off postconsent among women assigned to the hybrid IVR arm. Second, the low contact rates resulted in sample distortion for both modes. Third, hybrid IVR survey completers had higher odds of having secondary education than the CATI completers, indicating additional sample distortion for hybrid IVR surveys.

Therefore, we identified CATIs as a better approach for phone surveys in settings similar to Burkina Faso because of the less profound distortion of completers. Greater sample distortion among those randomized to the hybrid IVR survey is expected because the mode requires participants to answer the questionnaire without assistance, creating a higher cognitive burden on respondents than the CATI. Thus, responding is more difficult for those with lower education or who do not speak a majority language [18]. In this study, the response rate among women with no education randomized to the hybrid IVR survey was 12.3% compared with the response rate of 28.5% among women with a secondary or higher level of education. In the CATI survey, the response rates were comparable in all education groups.

This study illustrates that CATI phone follow-up surveys among women are feasible but suffer from noteworthy nonresponse. Failure to contact the sampled participants was the main cause of nonresponse, with 46% of hybrid IVR survey and 41% of CATI participants classified as noncontacts. Noncontact is also the main cause of nonresponse in high-income countries [8]. Refusal to participate was minimal in both arms. Finally, the hybrid IVR survey had increased nonresponse because of difficulties in responding to a phone keypad, with 3.5% of all eligible participants willing to participate but unable to navigate the phone keypad to begin the survey. Recent random digit dial (RDD) studies in the United States lost 9% [19] and 7% [18] of the sample when transferring respondents from the CATI to IVR survey. Among the hybrid IVR survey participants who were able to press 1 (and be transferred to IVR), there was a substantial break-off, with 20% answering less than half of the survey questions.

Studies using CATIs for remote data collection in Lebanon [20], Honduras, and Peru [21] have examined response rates and related sample distortion. These studies enrolled men and women during a baseline FTF survey who were recontacted using CATIs. The study in Lebanon had an 82% response rate but did not compare respondent and nonrespondent profiles [20]. The profile of completers in our study had a similar pattern as completers of phone surveys in Peru and Honduras, with the wealthy, educated, and urban also more likely to complete the phone follow-up [21]. The response rate at first contact was 33% in Peru and 59% in Honduras. However, these surveys, administered as part of a study conducted by the World Bank, found that young participants were more likely to complete the survey. Our results found an opposite association most likely because we enrolled only women [21]. We believe this is because gender norms result in different ownership and access in a context such as Burkina Faso. Young men may have the financial and social freedom to own a cell phone, whereas young women are less likely to be economically independent and to be given a phone by a parent, as their actions are more closely monitored. Female cell phone owners may still have less opportunity to use their phones compared with male cell phone owners because of the lack of resources to pay for communications and due to the possibility that women are more likely to have a gatekeeper to receive a phone call.

The study has notable strengths. The 2 modes used in our research, IVR and CATI, are rarely compared in low- or high-income countries [22]. Not only is the direct comparison of two remote modes rare but also it is uncommon for IVR studies in SSA to have a known sampling frame. Our sampling frame was a population-based FTF sample that allowed us to assess the characteristics related to completion, regardless of the woman’s participation in the phone follow-up. The FTF survey provided identical measures for phone survey respondents and nonrespondents, whereas many studies evaluating nonresponse error rely on surveys that do not have directly comparable indicators for nonrespondents. Knowledge of the characteristics of phone nonrespondents is also valuable for the survey design to oversample women who are less likely to respond and adjust remote data collection estimates through weighting. The study design also allows us to compare respondents with the full FTF sample (including women who do not own cell phones), where we see a dramatic difference in demographic profile. For example, among hybrid IVR survey completers, 54% of the population had a secondary education (data not shown), whereas only 19% of the full FTF female sample had a secondary education [14]. Finally, the randomization of respondents to CATIs or hybrid IVR surveys allows a more robust comparison of nonresponse by phone survey mode.

The follow-up design of the survey meant that women had already participated in a PMA2020 survey and consented to follow-up, yielding higher response rates than an RDD survey. Thus, our four survey outcomes rates must be interpreted with caution. Phone follow-up surveys conducted after an FTF survey in SSA have only investigated response rates among populations, including both male and female respondents, but have generally found higher response rates than those in our CATI survey [6]. Our CATI response rate was likely much lower because of the 11-month span between enrollment and follow-up and because women are harder to reach than men via cell phones [23]. Research from LMICs shows that rapid follow-up (defined as less than a month since the first contact) after enrolling in an FTF survey is key to reduce noncontact, the main cause of nonresponse in our survey [21,24]. Thus, our response rate highlights the difference in response rates when following up after a greater amount of time to create a new cross-sectional study originating from an FTF compared with a cohort quickly contacted for phone follow-up after an FTF interview. The response rate for the hybrid IVR survey was higher than that of other IVR surveys in SSA, most likely because of the selection of cell phone owners in our survey and because live interviewers introduced the survey. The only response rates available for comparison with our hybrid IVR survey rates are from surveys that use RDD sampling, not follow-up surveys, and use IVR without human introduction. Surveys in Mozambique and Zimbabwe had response rates of 9% and 8% [3,23], respectively, and a more recent RDD in Ghana had a response rate of 21% [12].

The context of Burkina Faso is unique in some areas pertinent to cell phone–based data collection, including high language fractionalization, making remote data collection more difficult because of language discordance between respondents and interviewers. Low female literacy reduces cell phone survey modes to those relying on interviewers. However, these contextual factors are similar across many West African countries.

The compound annual growth rate (CAGR) for cell phone ownership in SSA was in double digits at the beginning of the decade [1]. However, the CAGR is now half the previous level. Future cell phone ownership growth will be among rural and young people, but the time of rapid increase is waning: the percentage of cell phone owners in SSA is projected to only increase by 2% between 2023 and 2025 to 52% of the population [1]. Thus, the differences in phone owners and nonowners will not be quickly erased, and caution must be exercised when trying to create nationally representative estimates from phone surveys.

### Conclusions

We identified the characteristics related to CATI and hybrid IVR survey completion and concluded that among PMA2020’s target population, that is, women of reproductive age, CATI results in a more representative sample yet still greatly distorted sample. CATI and hybrid IVR survey completers were not reflective of the target population’s characteristics, in particular, education, geographic location, and age. This study is among the first to analyze phone survey nonresponse errors and to compare CATI and hybrid IVR surveys in SSA. Our results underscore that enrolling a representative sample of women in a phone survey in West Africa is challenging, regardless of cell phone mode, and should inform remote data collection efforts in West Africa.

## Abbreviations

AAPOR: American Association for Public Opinion Research
CAGR: compound annual growth rate
CATI: computer-assisted telephone interview
DHS: Demographic and Health Survey
FTF: face-to-face
IVR: interactive voice response
LMIC: low- and middle-income country
OR: odds ratio
PMA2020: Performance Monitoring and Accountability 2020
R4: round 4
RDD: random digit dial
SSA: sub-Saharan Africa
